# Surface Composition Impacts Selectivity of ZnTe Photocathodes in Photoelectrochemical CO_2_ Reduction Reaction

**DOI:** 10.1021/acsenergylett.4c02259

**Published:** 2024-12-09

**Authors:** Guosong Zeng, Guiji Liu, Gabriele Panzeri, Chanyeon Kim, Chengyu Song, Olivia J. Alley, Alexis T. Bell, Adam Z. Weber, Francesca M. Toma

**Affiliations:** † Liquid Sunlight Alliance, Lawrence Berkeley National Laboratory, 1 Cyclotron Road, Berkeley, California 94720, United States; ‡ Chemical Sciences Division, Lawrence Berkeley National Laboratory, 1 Cyclotron Road, Berkeley, California 94720, United States; § Department of Mechanical and Energy Engineering, Southern University of Science and Technology, Shenzhen 518055, China; ∥ Dipartimento di Chimica, Materiali e Ingegneria Chimica Giulio Natta, Politecnico di Milano, 20131 Milano, Italy; ⊥ Department of Chemical and Biomolecular Engineering, University of California Berkeley, Berkeley, California 94720, United States; # National Center for Electron Microscopy, The Molecular Foundry, Lawrence Berkeley National Laboratory, 1 Cyclotron Road, Berkeley, California 94720, United States; 7 Energy Technologies Area, Lawrence Berkeley National Laboratory, 1 Cyclotron Road, Berkeley, California 94720, United States; 8 Institute of Functional Materials for Sustainability, Helmholtz Zentrum Hereon, Kanstrasse 55, 14157 Teltow, Germany; 9 Faculty of Mechanical and Civil Engineering, Helmut Schmidt University, Hamburg 22043, Germany; 10 Department of Energy Science & Engineering, DGIST, Daegu 42988 South Korea

## Abstract

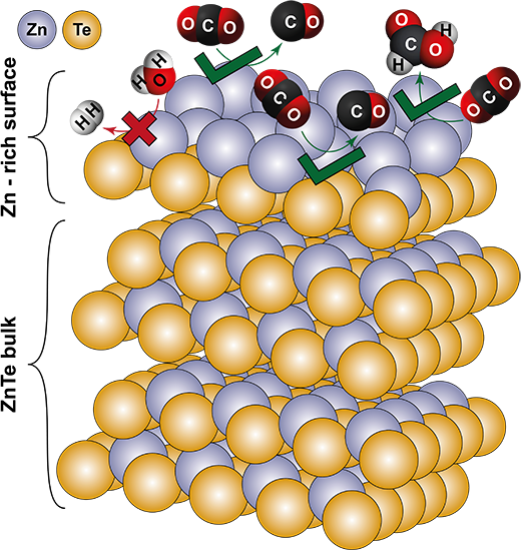

Light-driven reduction of CO_2_ into chemicals using a photoelectrochemical (PEC) approach is considered as a promising way to meet the carbon neutral target. The very top surface of the photoelectrode and semiconductor/electrolyte interface plays a pivotal role in defining the performance for PEC CO_2_ reduction. However, such impact remains poorly understood. Here, we report an electrodeposition-annealing route for tailoring surface composition of ZnTe photocathodes. Our work demonstrates that a Zn-rich surface on the ZnTe photocathode is essential to impact the CO_2_ reduction activity and selectivity. In particular, the Zn-rich surface not only facilitated the interfacial charge carrier transfer, but also acted as electrocatalyst for boosting carbon product selectivity and suppressing the hydrogen evolution reaction. This work provides a new avenue to optimize the photocathode, as well as improvement of the CO_2_RR performance.

Carbon neutrality is widely accepted as one of the main solutions to address contemporary climate change challenges. To achieve this goal, the utilization of solar energy has the potential to supplant the need for fossil fuels. However, significant limitations related to seasonal, regional, and diurnal fluctuations still hinder the widespread adoption of this source of energy. In this context, photoelectrochemical (PEC) CO_2_ reduction has attracted significant attention as a promising approach to store intermittent solar energy in fuels and chemicals as well as closing the chemical carbon cycle.^1−3^

In a typical PEC cell, photocathode materials can reduce CO_2_ to high-density carbon products. However, the CO_2_ reduction reaction (CO_2_RR) is a thermodynamically complex reaction, and viable photocathode materials for this process are also suitable for hydrogen evolution reaction (HER) and generally unstable, thus leading to either insufficient activity or selectivity, as well as to instability challenges for CO_2_RR.^4,5^ Among other candidates, zinc telluride (ZnTe) has recently gained increasing attention as a promising material for the CO_2_RR, due to its appropriate band gap (2.26 eV) for light harvesting, highly negative conduction-band-edge position suitable for the CO_2_RR, and predicted excellent durability in CO_2_RR.^6−9^ While these advantages make ZnTe a theoretically promising photocathode candidate for CO_2_RR, it has been reported that bare ZnTe photocathodes favor HER with ∼60% of Faradaic efficiency (FE) over CO_2_RR, thus resulting in low performance for light-driven CO_2_RR.^10,11^ Besides the specific thermodynamic and kinetic barriers of the material for CO_2_RR, the very top surface of the photoelectrode and semiconductor/electrolyte interface play a pivotal role in defining the performance for a given reaction, and this aspect is even more evident in complex reactions like CO_2_RR with multiple carbon product selectivity.^12^ To overcome these limitations, numerous reports have focused on adding mono- or multilayer catalysts on the ZnTe surface to provide additional catalytic active sites to boost selectivity to CO_2_RR.^13−16^ In contrast, the interface between the intrinsic ZnTe surface and electrolyte for PEC CO_2_RR remains largely unexplored. Further insight into interfacial kinetics between bare ZnTe and electrolyte can lead to fully exploiting the advantages of this material for CO_2_RR, and to further enhance the CO_2_RR performance of ZnTe/catalysts integrating systems.

In this work, we developed an electrodeposition-annealing route for tailoring the surface composition of ZnTe photocathodes. While we obtained pure phase ZnTe upon 380–550 °C annealing, we observed an interesting phenomenon that both activity and selectivity of ZnTe in PEC CO_2_RR vary with the annealing temperatures. The temperature-dependent behavior is attributed to surface-related properties that directly affect interfacial charge transfer during light driven CO_2_ reduction and determine the product selectivity. Specifically, the 550 °C annealing results in a Zn-rich region on the surface of ZnTe, which not only acts as a charge collector to accelerate photoelectron transfer and collection at the semiconductor/electrolyte interface but also adds active sites that favor the CO_2_RR and suppress competitive HER.

Polycrystalline ZnTe was electrodeposited on fluorine doped tin oxide (FTO)/glass substrate, in aqueous solution containing TeO_2_ and ZnSO_4_, adapted from a previously reported procedure.^10^ Additionally, we stabilized Te^4+^ species by complexation with citrate ions in the plating bath.^17^ This process allows for controlled diffusion of the Te^4+^ complexed species to the working electrode surface, where the Te^4+^ discharge and react with Zn^2+^ species to form ZnTe. X-ray diffraction (XRD) shows poor crystallinity of the as-deposited ZnTe with Te as an impurity (Figure 1a). After annealing at 300 °C under N_2_ atmosphere, the XRD pattern of ZnTe annealed sample exhibits the characteristic (111), (200), (220), and (311) reflections (JPCDS PDF# 15–0746, cubic structure), while the Te impurity is still present.^18,19^ At 380–550 °C annealing, polycrystalline, phase-pure ZnTe can be obtained. The ratios of peak intensity for (111)/(220), and (111)/(311) were similar for ZnTe thin films annealed under 380 to 550 °C, indicating that the (111) facet was dominant independent of the annealing temperature within this temperature range. Annealing at temperatures higher than 600 °C causes partial evaporation of ZnTe from the substrate (Figure S1). Moreover, the surface morphology of the as-deposited and annealed ZnTe (Figure 1b and c, Figure S2) showed similar globular polycrystalline structures by scanning electron microscopy (SEM) and atomic force microscopy (AFM, Figure S1), which is consistent with previous reports.^10^

**Figure 1:**
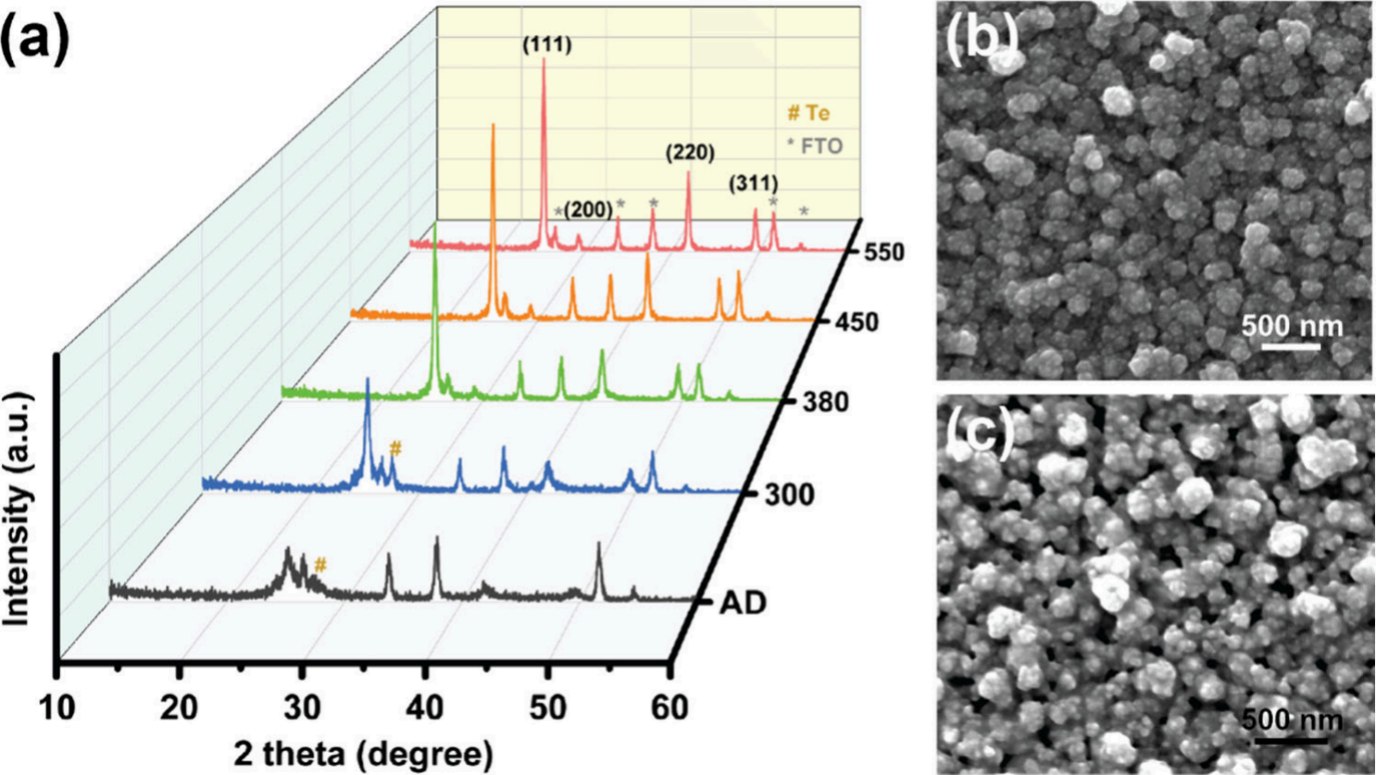
(a) XRD patterns as-deposited (AD) ZnTe and of ZnTe thin films annealed under various temperature; (b, c) SEM images of ZnTe thin films annealed under 380 and 550 °C, respectively.

In contrast to the XRD and SEM results, which are relatively consistent across all the ZnTe samples annealed at 380–550 °C, the ZnTe samples with varied annealing temperature exhibit intriguingly different PEC behaviors toward the CO_2_RR. Compared to ZnTe under 380 °C (noted as ZnTe-380), the ZnTe annealed at 550 °C (noted as ZnTe-550) displays significantly improved photocurrent over the entire operating potential range, reaching a photocurrent density of −5 mA/cm^2^ at −0.8 V vs reversible hydrogen electrode (RHE). Moreover, both ZnTe samples annealed at 380 and 550 °C exhibit excellent stability over 2 h (Figure 2b and Figure S3). To verify that the observed stable photocurrent of ZnTe photocathodes stems from catalytic activity toward the CO_2_RR, the evolved gaseous products and liquid products have been quantified by gas chromatography and high-performance liquid chromatography, respectively. Interestingly, ZnTe-550 showed enhanced selectivity toward CO_2_RR products by 2 times, reaching 60% of FE for C_1_ products (including 45% CO and 15% formic acid), accompanied by significant decrease of HER from 60% down to 30%, with respect to ZnTe-380 (Figure 2c). In addition to FE, to understand the intrinsic activity toward each product, we further plotted partial photocurrent density for ZnTe-550 and ZnTe-380 (Figure S4). These data support that the suppressed HER as well as improved C_1_ production is the origin of the enhanced selectivity toward CO_2_RR. Notably, the selectivity of CO_2_RR products on ZnTe-550 surpasses the state-of-the-art ZnTe photocathodes for CO_2_RR, which showed only 30% of selectivity to CO_2_RR products using bare ZnTe.^15,20^

**Figure 2:**
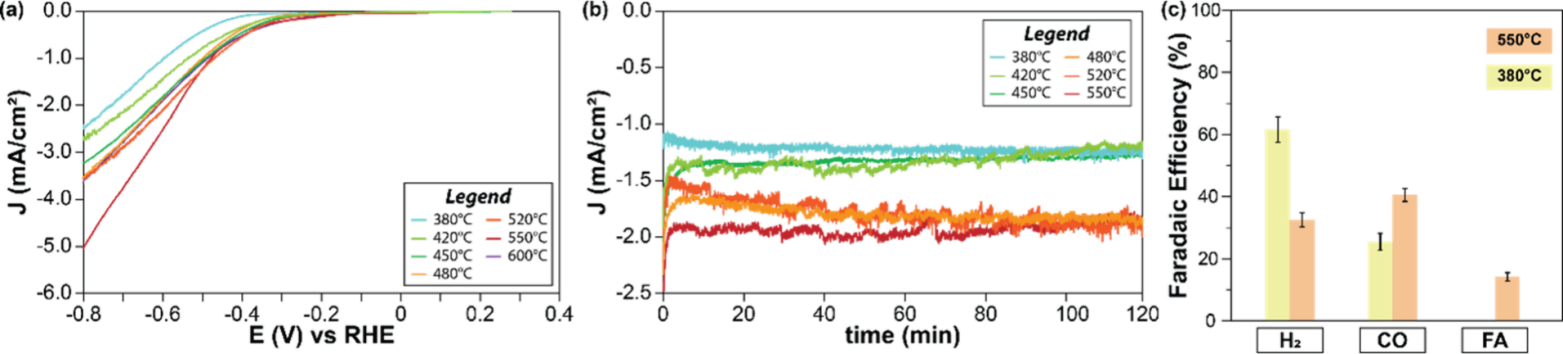
(a) *J*–*V* curves of ZnTe thin films after annealing under different temperature in 0.1 M KHCO_3_ (CO_2_ saturated) aqueous solution (pH = 6.8) under AM 1.5 G simulated sunlight (100 mW cm^–2^); (b) Chronoamperometry (CA) of ZnTe photocathodes after annealing under different temperature at −0.6 V_RHE_ under AM 1.5 G simulated sunlight (100 mW cm^–2^) in 0.1 M KHCO_3_ (CO_2_ saturated) aqueous solution (pH = 6.8); (c) Faradaic efficiencies of H_2_, CO and formic acid for ZnTe-380 and ZnTe-550 at −0.6 V_RHE_ under AM 1.5 G simulated sunlight (100 mW cm^–2^) in 0.1 M KHCO_3_ (CO_2_ saturated) aqueous solution (pH = 6.8). Data are presented as mean values based on triplicates.

It is generally acknowledged that the observed photocurrent is a result of light absorption, bulk charge transport and interfacial charge transfer.^21^ To look into the origins of this temperature-dependent photocurrent, we examined light absorption properties of ZnTe-550 and ZnTe-380 by UV–vis spectroscopy and incident photon to current conversion efficiency (IPCE) measurements. The UV–vis spectra show almost identical light absorbance for both ZnTe-550 and ZnTe-380 (Figure S5). Furthermore, the ZnTe-550 shows superior IPCE values over the entire range of wavelengths, compared to ZnTe-380 (Figure S6), which aligns well with the LSV results (Figure 2a). It is also worth noting that the onset wavelength of photocurrent response is around 550 nm for both ZnTe-550 and ZnTe-380, which is close to the absorption edge of ZnTe. Thus, the changes in the photocurrent cannot be attributed to changes in the band gap of the material or presence of midgap states.

We next examine the bulk charge transport property of ZnTe-550 and ZnTe-380 by measuring the photocurrent of ZnTe-550 and ZnTe-380 in the presence of an Fe(CN)_6_^(3–/4−)^ redox couple. The Fe(CN)_6_^(3–/4−)^ redox couple ensures the collection of all charge carriers reaching the semiconductor–electrolyte junction, thereby allowing an assessment of bulk charge transport without influence from surface electrocatalytic process.^22^ It appears that ZnTe-550 only shows slightly better photocurrent than ZnTe-380, in contact with the Fe(CN)_6_^(3–/4−)^ redox couple (Figure S7). Taken together, neither bulk light absorption nor charge transport is distinctively different between ZnTe-550 and ZnTe-380, leaving interfacial charge transfer as the vital point in determining the PEC performance for the CO_2_RR.

To investigate the interfacial charge transfer, electrochemical impedance spectroscopy (EIS) was carried out for ZnTe-550 and ZnTe-380 (Figure S8). The most remarkable change in the EIS results is the significant decrease in interfacial resistance at the electrode/electrolyte for ZnTe-550, with respect to ZnTe-380. This result is supportive of a facilitated electron transfer at the electrode/electrolyte for ZnTe-550 and can explain the increase of the photocurrent in Figure 2a.

To provide further insights into the charge-transfer mechanism, photoconductive atomic force microscopy (pc-AFM) can provide information about photocurrent heterogeneity on the nanoscale. Although the tip–sample interactions at the solid/solid interface are different from the solid/liquid interface during water splitting or CO_2_ reduction conditions, the correlation between photocurrent distribution and the specific feature at nanoscale can still provide relevant information between the improvement of the photocurrent generation and morphology. As a result, pc-AFM has been extensively used in the studies of the PEC water splitting and CO_2_ reduction reactions.^23−28^ Therefore, we performed pc-AFM measurements on ZnTe-550 and ZnTe-380 in the dark and under illumination. The comparable results in surface topography and roughness (Sa ∼ 22–26 nm) of ZnTe upon 380–550 °C annealing rule out the role of surface morphology or roughness for the different PEC performance (Figure 2b and Figure S2).^29^ However, the photoresponses of these two surfaces exhibited very different behaviors. As shown in Figure 3, under illumination with an applied bias of 300 mV, pc-AFM reveals an increase in the photocurrent of more than one order of magnitude in favor of the ZnTe-550 sample (500 pA) as compared to the ZnTe-380 sample (34 pA). Figure S9 shows the pc-AFM dark current and photocurrent for the two samples. Interestingly, differences between changes in the current measured without illumination under the same bias for the ZnTe-550 was much less noticeable, while the dark current for the ZnTe-380 was half of the measured current under illumination (Figure S9c and S9f). These pc-AFM results validate that the ZnTe-550 surface has a much improved charge carrier transfer capability when compared to ZnTe-380, which is fully in line with the EIS results discussed earlier.

**Figure 3:**
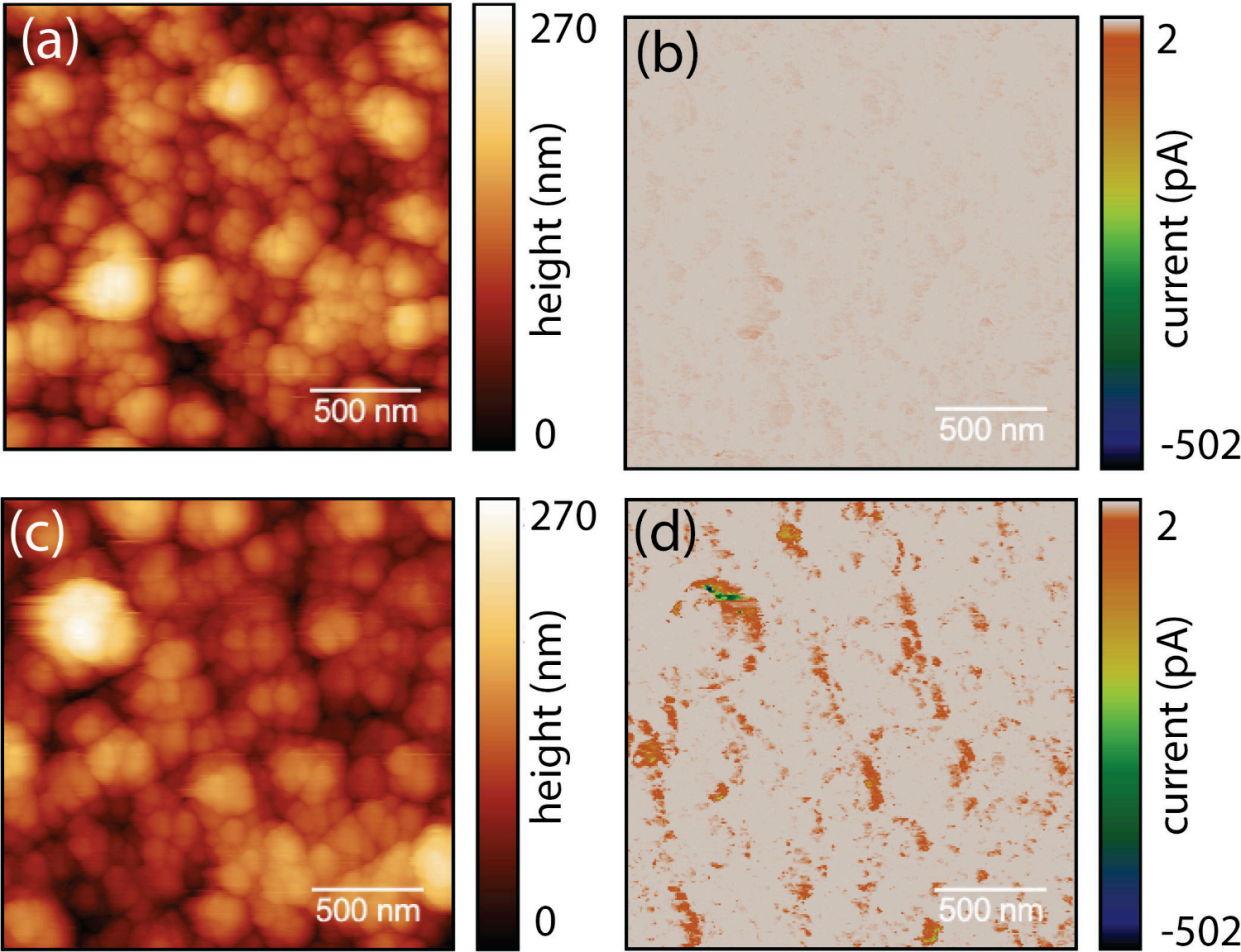
pc-AFM analysis of ZnTe thin films under 380 and 550 °C: (a) Analyzed area of ZnTe-380 surface. (b) Corresponding photocurrent measured from the ZnTe-380 surface. (c) Analyzed area of ZnTe-550 surface. (d) Corresponding photocurrent measured from the ZnTe-550 surface.

To further understand the relationship between the surface and performance, we examined the surface composition of the ZnTe samples by scanning transmission electron microscopy (STEM) with electron energy loss spectroscopy (EELS). STEM images show that the grain shapes and sizes of ZnTe-380 and ZnTe-550 are identical (Figure 4a and 4d), which is consistent with AFM and SEM results. However, EELS line scans suggest that the Zn and Te are homogeneously distributed within ZnTe-380 (Figure 4b), while there existed a Zn rich region at the outer surface of ZnTe-550, i.e., Zn reached its maximum intensity at the position around 10 nm, whereas the intensity of Te was still ramping up and reached its maximum value 5 nm later at the position of 15 nm (Figure 4e). To confirm the findings through EELS, X-ray photoelectron spectroscopy (XPS) and ion scattering spectroscopy (ISS) were also performed on both the ZnTe-380 and ZnTe-550 surfaces. The ISS spectra show that the Zn signal on the outer surface of ZnTe-550 is significantly higher than that of ZnTe-380, in excellent agreement of EELS results (Figure S10). The Zn LMM Auger peak and Te 3d core level obtained by XPS further revealed that, besides the dominant Zn–Te peak and Na KLL Auger peak (presumably originated from the trisodium citrate during electrodeposition), both ZnTe-380 and ZnTe-550 surfaces contained Zn^0^ and Te^0^, while the Zn^0^ from ZnTe-550 is more significant than ZnTe-380 (Figure 4c, 4f and Figure S11).^14,30−32^ Given that the penetration depth of XPS is about 7–9 nm, and the Auger signal is even more surface sensitive, we can confirm that the change of the material structure and composition mainly occurs in the first few nanometer region. This finding also explains the similar bulk material properties observed in Figure 1, while obvious enhancements are found in Figure 2 and Figure 3. Correlating all the results obtained by various characterization techniques, we conclude that the Zn-rich region on the surface of ZnTe-550 plays a key role in accelerating photoelectron transfer and collection at the surface. Recent theoretical simulation revealed that Zn contributed more in the conduction band of ZnTe than in the valence band.^33^ Accordingly, a Zn-rich surface may facilitate electron transfer, which is in good agreement with our experimental findings.

**Figure 4:**
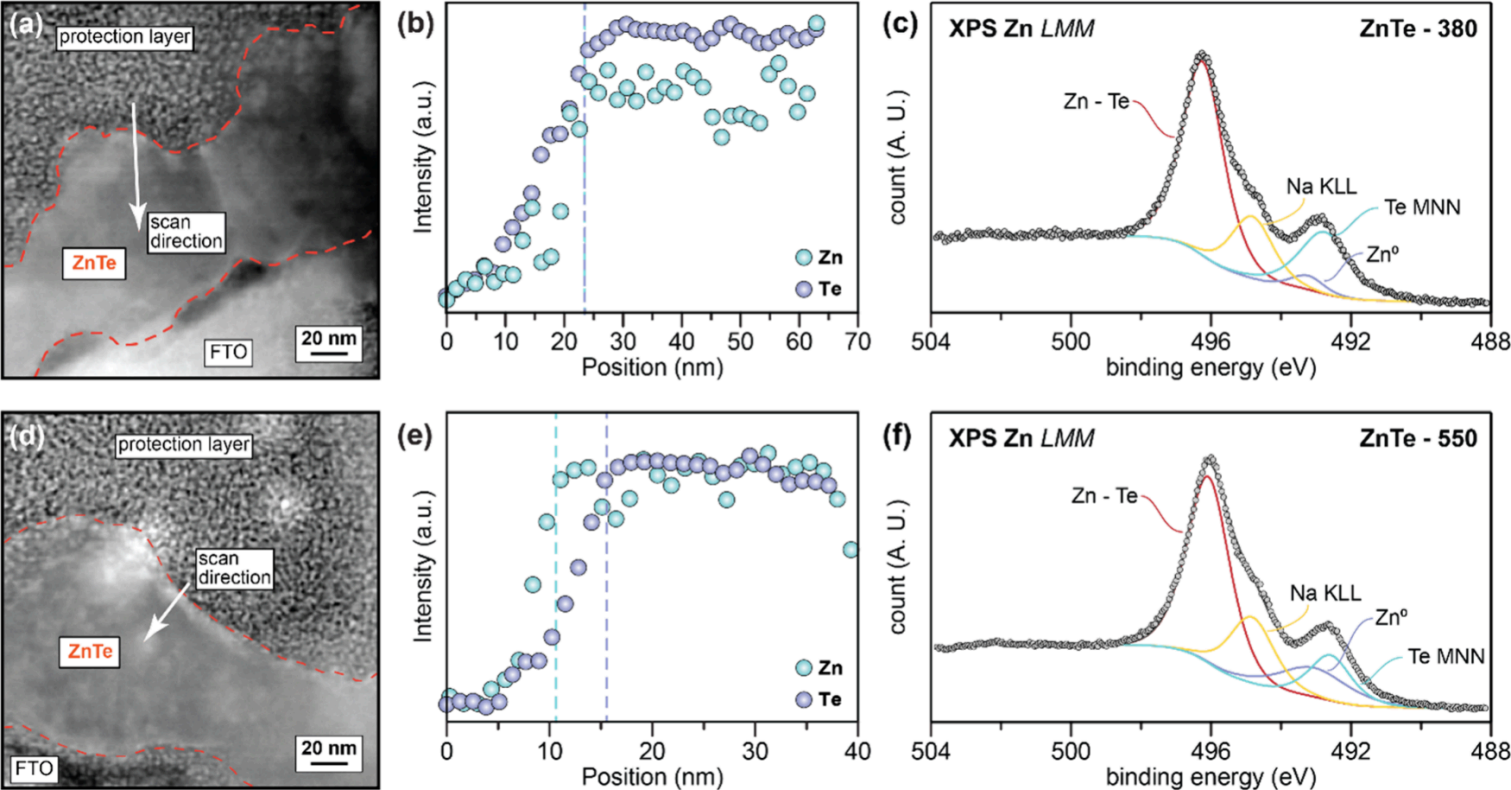
STEM/EELS measurements of ZnTe thin films: (a,b) ZnTe-380, (d,e) ZnTe-550; XPS analysis of ZnTe thin films: (c) ZnTe-380, (f) ZnTe-550.

Furthermore, Zn is known as an efficient catalyst for electrochemically reducing CO_2_ into CO and formic acid.^34−36^ In this work, the 550 °C annealing induced a Zn-rich surface and effectively enhanced the charge transfer at the solid/liquid interface. Accordingly, we surmise that the Zn-rich region also acts as a catalytic site for directing photogenerated charge carriers for desired CO_2_ reduction at the semiconductor/electrolyte interface. To validate this hypothesis, we performed CO_2_RR in the dark using ZnTe-380 and ZnTe-550 (Figure S12). As expected, the FE values for H_2_ and C_1_ products on ZnTe-550 and ZnTe-380 in the dark exhibited a very similar trend to those observed for ZnTe-550 and ZnTe-380 under light. The HER was suppressed, giving rise to higher C_1_ product generation. These results further verify that the Zn-rich surface not only facilitates the charge transfer but also acts as an electrocatalyst that enhances the selectivity of the CO_2_RR to carbon products.

In this work, we reported a simple annealing method to effectively modify the surface of ZnTe, resulted in an improved interfacial charge transfer toward PEC CO_2_RR. By employing state-of-the-art photoconductive AFM, STEM–EELS, and XPS characterization techniques, we showed the presence of a Zn-rich region on the surface of ZnTe upon 550 °C annealing, which not only acts as a charge collector to accelerate photoelectron transfer and collection at the semiconductor/electrolyte interface, but also plays as a catalyst, directing photoelectrons into CO_2_RR and suppressing competitive HER. This work proves the fact that, before adding electrocatalysts, there is still sufficient room for the optimization of thermodynamically viable materials.^24^ The results of our work highlight the importance of surface compositions of photocathodes on the observed PEC CO_2_RR activity and selectivity. Such knowledge can improve the development of active and selective photocathodes and provide further insights into the reaction mechanism of light driven CO_2_RR.

## Supporting Information Available

List of utilized materials and chemicals, as well as the details of the experimental procedures; supplementary data about the visual appearance of the samples, AFM images, chronoamperometry measurements, partial photocurrent densities, UV–vis spectra, IPCE data, experiment with redox couple, EIS measurements and analysis, ISS and XPS spectra, AFM images, and partial current densities (PDF)
All data used to plot the figures reported in Figure 1a, 2a–c, 4b–c, and 4e–f (XLSX)
