# Supplementary material for: Surface Composition Impacts Selectivity of ZnTe Photocathodes in Photoelectrochemical CO2 Reduction Reaction
Source: ACS Energy Lett. 2024 Dec 9;10(1):34–9. doi: 10.1021/acsenergylett.4c02259 (PMC11731327; doi:10.1021/acsenergylett.4c02259)
Supplement: Supplementary file 1 — nz4c02259_si_001.pdf [file nz4c02259_si_001.pdf]

Supporting Information

Surface composition impacts selectivity of ZnTe photocathodes in photoelectrochemical CO_2 reduction reaction

Guosong Zeng[1, 2, 3], Guiji Liu[1, 2], Gabriele Panzeri[2, 4], Chanyeon Kim[1,2,5,6], Chengyu Song[7], Olivia J Alley[2], Alexis T. Bell[1,2,5], Adam Z. Weber[1,8], and Francesca M. Toma[1, 2, 9,10*]

1. Liquid Sunlight Alliance, Lawrence Berkeley National Laboratory, 1 Cyclotron Road, Berkeley, California 94720 United States.

2. Chemical Sciences Division, Lawrence Berkeley National Laboratory, 1 Cyclotron Road, Berkeley, California 94720 United States.

3. Department of Mechanical and Energy Engineering, Southern University of Science and Technology, Shenzhen, 518055, China.

4. Dipartimento di Chimica, Materiali e Ingegneria Chimica Giulio Natta, Politecnico di Milano, 20131 Milano, Italy.

5. Department of Chemical and Biomolecular Engineering, University of California Berkeley, Berkeley, California 94720 United States.

6. Department of Energy Science & Engineering, DGIST, Daegu 42988 South Korea

7. National Center for Electron Microscopy, The Molecular Foundry, Lawrence Berkeley National Laboratory, 1 Cyclotron Road, Berkeley, California 94720 United States.

8. Energy Technologies Area, Lawrence Berkeley National Laboratory, 1 Cyclotron Road, Berkeley, California 94720 United States.

9. Institute of Functional Materials for Sustainability, Helmholtz Zentrum Hereon, Kanstrasse 55, 14157, Teltow, Germany.

10. Faculty of Mechanical and Civil Engineering, Helmut Schmidt University, Hamburg 22043, Germany.

*Corresponding Author: Francesca.Toma@hereon.de

Materials and Methods

ZnTe thin film synthesis

Polycrystalline ZnTe was electrodeposited on fluorine doped tin oxide (FTO)/glass substrate (TEC-7 Sigma Aldrich), adapted from a previously reported procedure (J. Mater. Chem. A 2015, 3, 1089–1095). In a typical experiment, 0.25 mM tellurium dioxide (Sigma Aldrich, ⩾99%) solution was dissolved in 30 mL sulfuric acid (1M, VWR chemicals, 95-98%). Then 20 mM zinc sulfate monohydrate (Sigma Aldrich, ⩾99.9%) and 100 mM trisodium citrate dihydrate (Sigma Aldrich) were added in the solution. In addition, 3 M sodium hydroxide (Sigma-Aldrich, ⩾98%) was added to the solution until the pH of the plating solution reached 4. ZnTe as deposited on the substrate with a constant potential: -0.65 V vs. Ag/AgCl in a three-electrode configuration at 50 ℃ for 2 h. After deposition, the samples were gently rinsed with water and then dried with a nitrogen gun.

To guarantee that ZnTe has not been affected by the FTO when annealed at its maximum working temperature, additional ICP-MS was performed. The ZnTe was totally dissolved in nitric acid and the solution was analyzed by ICP-MS. The results showed that the Sn concentrations for both 380 °C annealed ZnTe and 550℃ annealed ZnTe were lower than 50 ppb, which was already near the detection limit of ICP-MS, demonstrating that 550 ℃ high temperature doesn't cause any potential ion migration to the ZnTe thin film. Meanwhile, the FTO substrate was also confirmed with no change of the resistivity after annealed under 550 ℃.

Photoelectrochemical testing

The photoelectrochemical (PEC) tests were performed with front illumination of the ZnTe photocathode. Both linear sweep voltammetry (LSV) and chronoamperometry (CA) were performed in a glass cell filled with 0.1M $KHCO_3$ (CO_2 saturated) aqueous solution (pH = 6.8) under AM 1.5G simulated sunlight (100 mW cm^{-2}). The scan range of LSV was set to be from open circuit potential (E_{OC}) to 0.8 V vs RHE, and the CA was carried out at a constant bias of 0.6 V vs RHE. Ag/AgCl was used as the reference electrode and a Pt wire was used as the counter electrode. The temperature of the electrolyte was monitored through the 2 hours long-term stability test and it was within 24 – 28 ℃.

X-ray photoelectron spectroscopy

Surface chemical composition of ZnTe was obtained by a laboratory XPS (Kratos Axis Ultra DLD system). 1,486.6 eV Al Kα source was used as the incident beam to excite the core-level electrons from the sample. Pass energy was set to 20 eV, and 0.05 eV was used as scan step size. The spectral fitting was conducted using CasaXPS analysis software. The binding energy scales of all core levels were corrected to the Te 3d of Te-Zn bond at 572.5 eV.

Scanning transmission electron microscopy (STEM) / Electron energy loss spectroscopy (EELS)

STEM and EELS characterizations were performed on an FEI F20 UT Tecnai microscope at the National Center for Electron Microscopy, Lawrence Berkeley National Laboratory, using an accelerating voltage of 200 kV. STEM-EELS probe size is 0.2 nm. convergent angle is 15 mrad, and EELS collection angle is 42 mrad.

X-ray diffraction

XRD was conducted on an X-ray diffractometer (SmartLab, Rigaku) using Cu Kα X-ray source.

Scanning electron microscopy

SEM images were obtained using Quanta FEG 250, FEI.

Photoconductive atomic force microscopy

pc-AFM analysis was performed on ZnTe thin film samples with Bruker Dimension Icon. PeakForce TUNA mode was used to for the morphology and current measurements simultaneously. A PtIr probe (spring constant of 2.8 Nm^{-1}) was used for the scanning. A white light source with a grazing incident angle was used to illuminate the surface during the acquisition.

UV-vis

Transmission and spectral reflectance measurements were performed on a Shimadzu SolidSpec-3700 UV/Vis/NIR spectrometer using an integrating sphere.

Incident photon-to-current efficiency (IPCE) measurements

IPCE measurements were carried out using a Newport 300 W Ozone free Xe lamp where the optical output was passed through an Oriel Cornerstone 130 1/8m monochromator. The sample

current was measured with a Gamry Reference 600 potentiostat. ZnTe photocathodes were configured as the working electrodes, a coiled Pt wire as the counter electrode, and an Ag/AgCl (3 M NaCl, BASI) as the reference electrode. Measurements were performed in 0.1 M $KHCO_3$ electrolyte saturated with CO_2 (pH 6.8) at an applied bias of -0.6 vs. RHE. The monochromatic light was stepped in 10 nm intervals and chopped at a period of 8 seconds in which the sample was illuminated only in the first 4 seconds of each period. The current under illumination was calculated by averaging readings in the last second of the illuminated interval. The dark currents were calculated by averaging the last second of the non-illuminated interval in every period. The photocurrent was calculated by reducing the dark current from the current under illumination at each cycle. The incident optical output at each wavelength was measured with a Newport 71648 photodiode.

Product Analysis

Gaseous products formed by PEC CO_2RR were separated from liquid electrolysis in a gas-tight reservoir and then analyzed by online gas chromatograph (GC, 7890B, Agilent) equipped with a pulsed-discharge helium ionization detector and ShinCarbon ST and Hayesep-Q capillary columns (Agilent); helium (99.9999%, Praxair Inc.) was used as the carrier gas. For quantitative analysis, a calibration curve for each gaseous product was generated by measuring the signal for each component obtained by analysis of a series of NIST-traceable standard gas mixtures (100 to 8000 ppm, Airgas Inc.). Liquid products collected in the catholyte reservoir over a period of 30 min were analyzed in a high-pressure liquid chromatograph (HPLC) (UltiMate 3000, Thermo Scientific) equipped with Aminex HPX 87-H columns (Bio-Rad Inc.) and a refractive index detector (RID). The signal for each liquid product was quantified using a calibration curve based on a series of standard solutions for each product in the range of concentration from 0.1 to 20 mM. Faradaic efficiency (FE) was calculated as $F=(nFC_iV)/I_{total}$ *100%, where n is the number of electrons transferred, F is Faraday's constant, C_i is molar concentration of species i, V is the total volumetric flow rate and I_{total} is the measured total current.

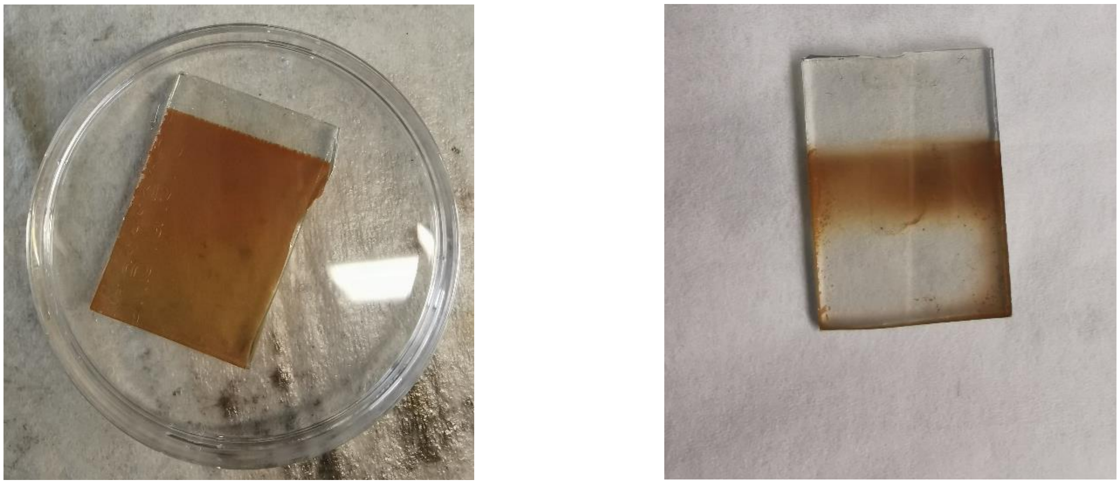

Fig. S1 (left) ZnTe under 550 °C; (right) ZnTe under 600 °C.

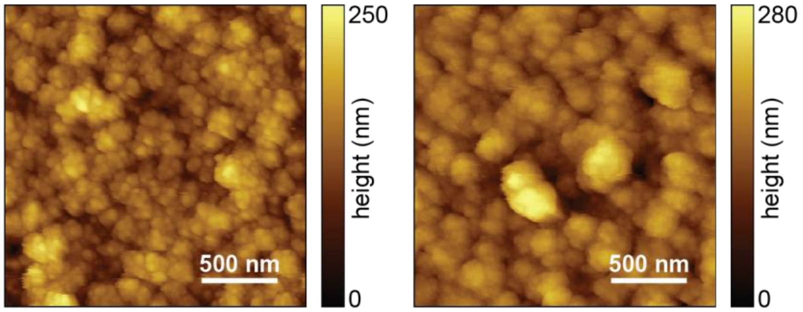

Fig. S2 AFM images of ZnTe thin films annealed under 380 °C and 550 °C, respectively.

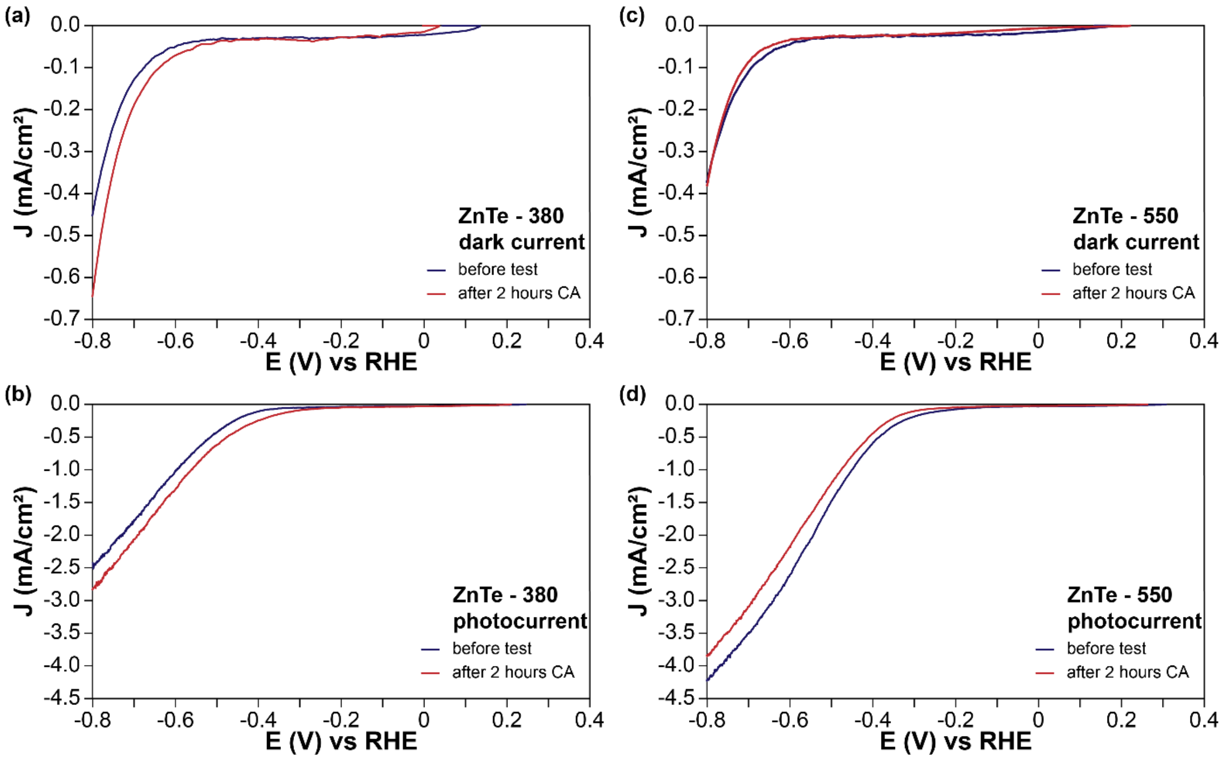

Fig. S3 Photocurrent and dark current of both ZnTe-380 and ZnTe-550 before and after 2-hours CA testing.

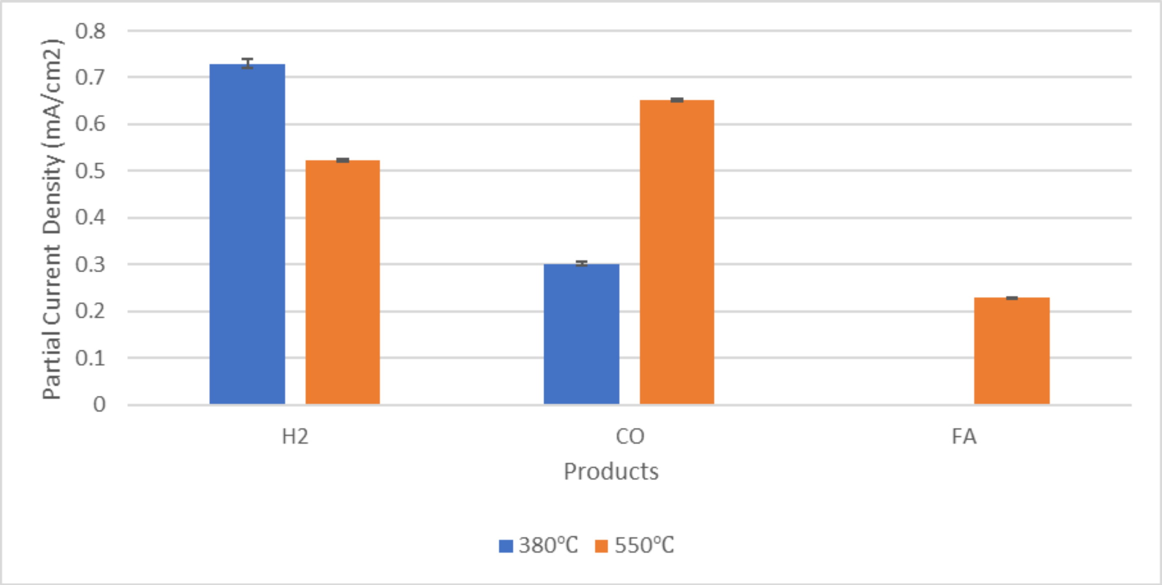

Fig. S4 Partial current density of H_2, CO and formic acid for ZnTe-380 and ZnTe-550 at -0.6 V_{RHE} in dark in 0.1 M $KHCO_3$ (CO_2 saturated) aqueous solution (pH = 6.8).

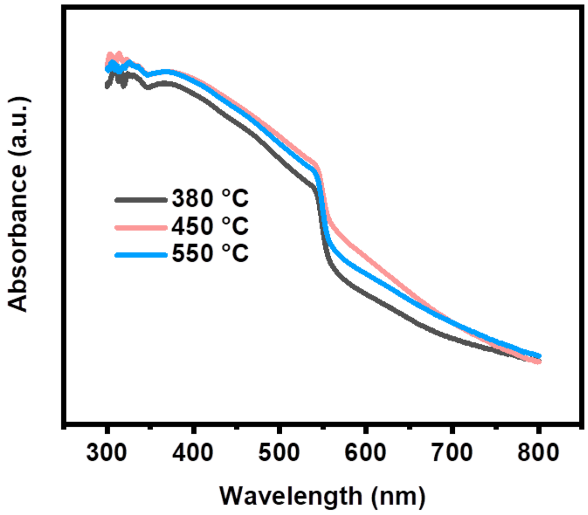

Fig. S5 UV-vis of ZnTe thin films annealed under different temperature.

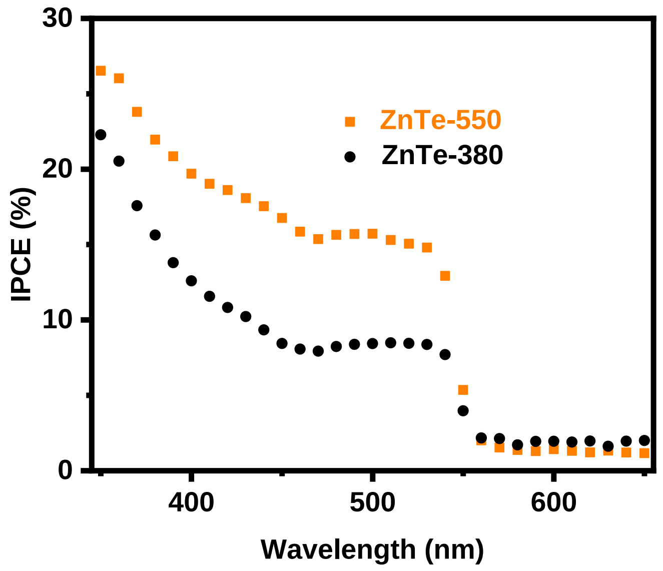

Fig. S6 IPCE values of ZnTe after annealing at 380 and 550 °C in 0.1 M $KHCO_3$ solution (CO_2 saturated) at -0.6 V vs. RHE.

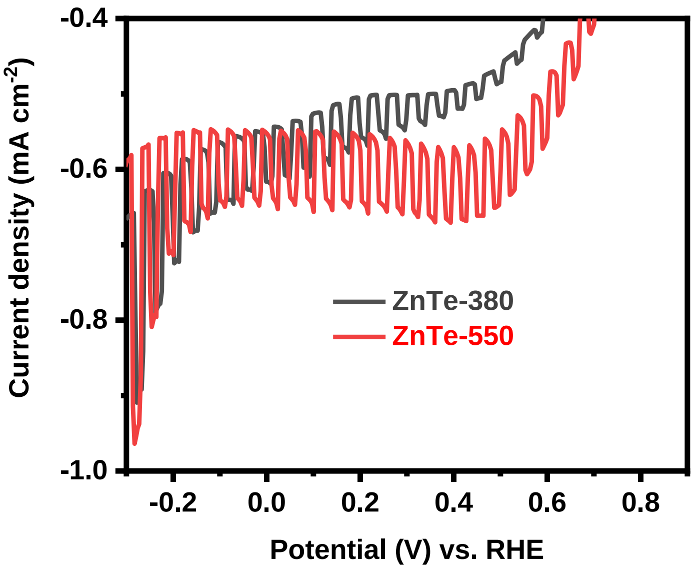

Fig. S7 J-V curves of ZnTe after annealing at 380 and 550 °C in contact with 5 mM $K_4Fe(CN)_6$ / 20 mM $K_3Fe(CN)_6$ redox couple.

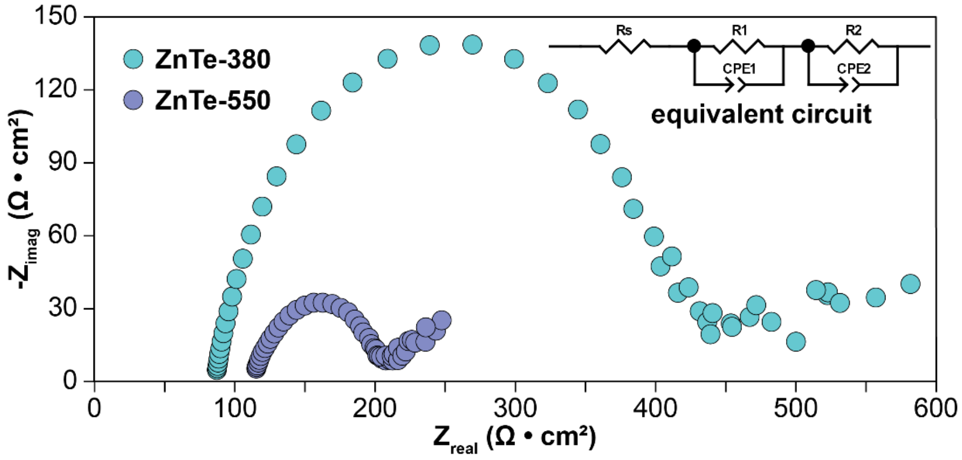

Fig. S8 EIS and equivalent circuit of ZnTe-380 and ZnTe-550. The EIS results were fitted into the models of two RC circuits. The values of resistances (R) and constant phase elements (CPE) as a result of fitting are presented in Table-S1. The RC circuit with the largest resistance (R2/CPE2) usually represents electrode/electrolyte interface, while R1/CPE1 is related to the electron transport inside the electrode (Int. J. Hydrogen Energy 2011, 36, 9462– 9468; J. Am. Chem. Soc. 2013, 135, 14, 5375–5383).

Table-S1: Resistance comparison of ZnTe-380 and ZnTe-550

Element	380°C	550°C
R_s	86.69	107
R_1	465.6	224
CPE 1	25×10^{-5}	8.9×10^{-5}
R_2	**1881**	**426.3**
CPE 2	1.3624×10^{-5}	5.8541×10^{-5}

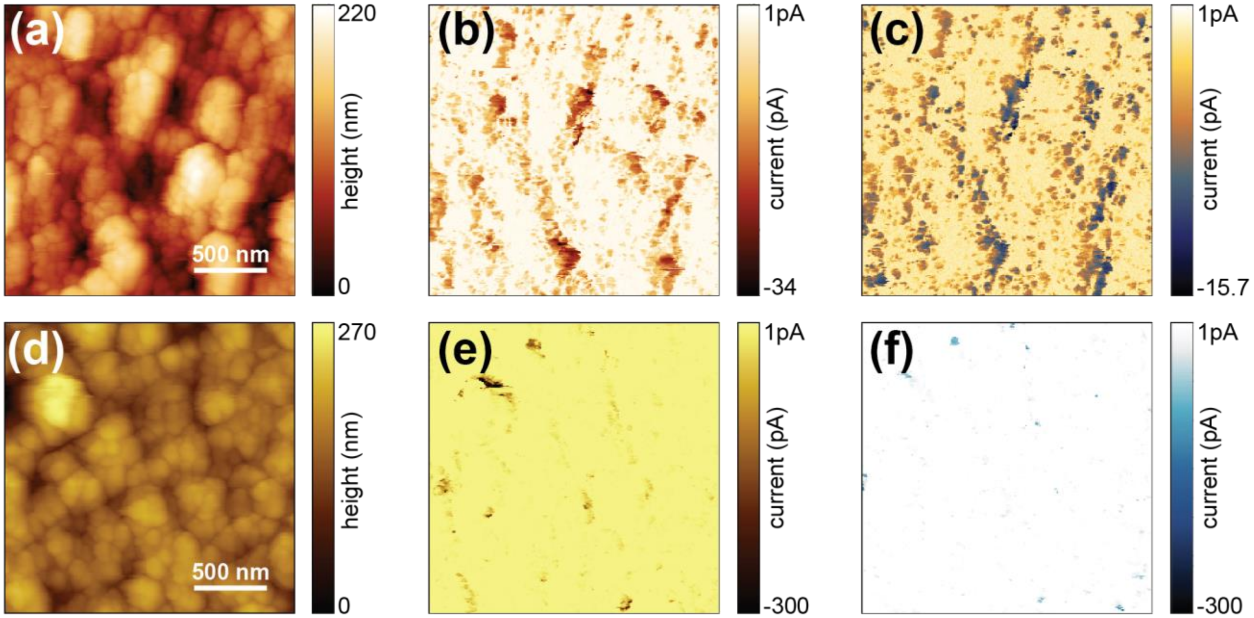

Fig. S9 (a) morphology of ZnTe-380; (b)photocurrent of ZhTe-380 at same location acquired by pc-AFM under illumination and 300 mV bias; (c) dark current of ZhTe-380 at same location acquired by c-AFM under 300 mV bias; (d) morphology of ZnTe-550; (b)photocurrent of ZhTe-550 at same location acquired by pc-AFM under illumination and 300mV bias (color bar set to be the same scale as ZnTe-550 in dark for straight comparison); (c) dark current of ZhTe-550 at same location acquired by c-AFM under 300mV bias.

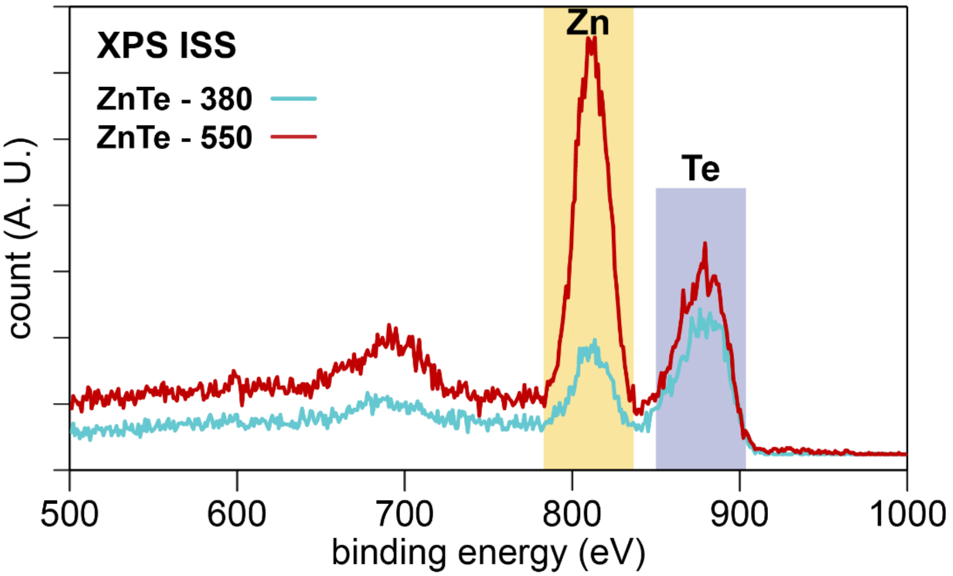

Fig. S10 ISS of ZnTe-380 and ZnTe-550.

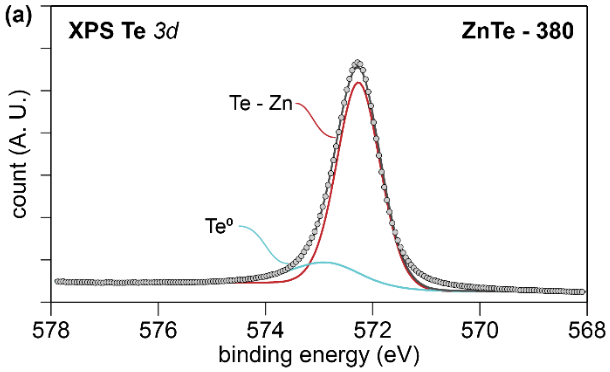

Fig. S11 XPS Te 3d core levels of ZnTe-380 and ZnTe-550.

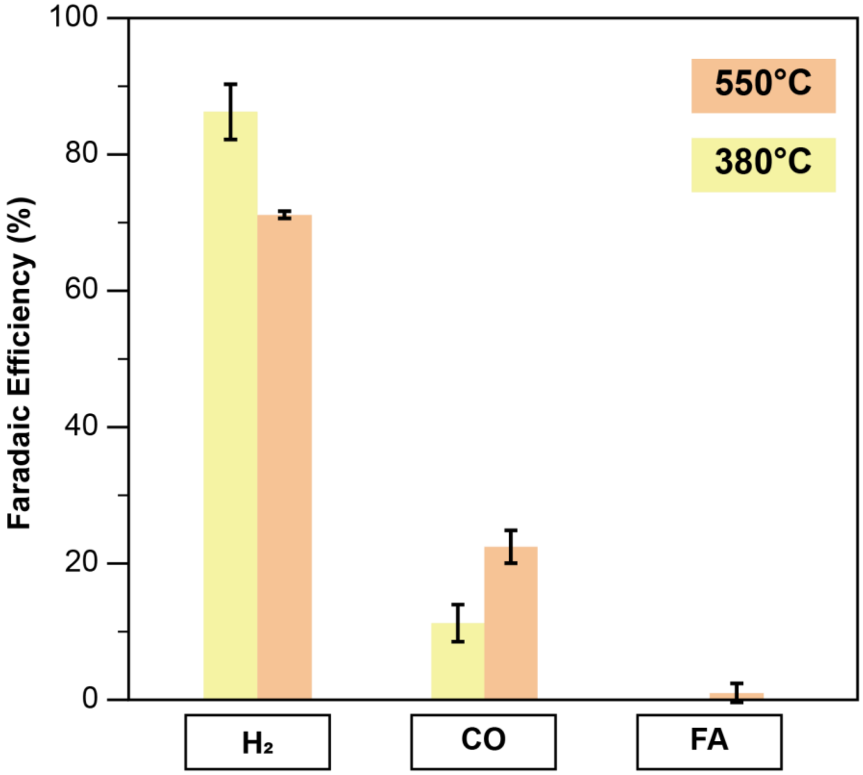

Fig. S12 Faradic efficiencies of H_2, CO and formic acid for ZnTe-380 and ZnTe-550 at -0.6 V_{RHE} in dark in 0.1 M $KHCO_3$ (CO_2 saturated) aqueous solution (pH = 6.8).
